# Cancer: Dairy Paradox

**DOI:** 10.1289/ehp.116-a114b

**Published:** 2008-03

**Authors:** M. Nathaniel Mead

The etiology of colorectal disease revolves around genetic and environmental factors, particularly diet. A meta-analysis in the 7 July 2004 issue of the *Journal of the National Cancer Institute* suggested that consuming more dairy products and calcium may reduce colorectal cancer risk, but epidemiologic studies on this link have yielded inconsistent results. One explanation for this inconsistency may be the timing of exposure: cancer develops over decades, and early-life exposures to carcinogens and growth factors could be a critical factor. A new study designed to address this possibility has found that adults who consumed more dairy during childhood may have a greater risk of developing colorectal cancer in adulthood. The results appear in the December 2007 issue of the *American Journal of Clinical Nutrition*.

Colorectal cancer is among the leading causes of mortality in developed countries; according to the National Cancer Institute, about 630,000 deaths were expected to occur worldwide in 2007. Even moderate changes in diet and lifestyle could prevent at least 70% of all colorectal cancer cases, according to a review in the December 2002 *Gastroenterology Clinics of North America*. The main culprits appear to be excessive caloric intake, as well as frequent consumption of red meat, processed meats, alcohol, and refined carbohydrates.

The historical cohort study employed data from the Carnegie Survey, conceptualized by Sir John Boyd Orr, which recorded food consumption patterns in 1,343 English and Scottish families from 1937 to 1939. The survey was designed to investigate the long-range impact of children’s diet, growth, living conditions, and health on adult cardiovascular disease.

Dietary data were obtained using a 7-day household inventory; also, weighed inventories of all foods in the household were conducted at the start and end of the survey period. The average follow-up time for adults included in this study was 65 years; 4,374 individuals were available for follow-up between 1948 and 2005. Daily intake of dairy products ranged from less than 0.5 cup at the lowest level to nearly 2 cups at the highest; liquid milk constituted 94% of the dairy intake.

Those individuals who grew up in families reporting the highest levels of dairy consumption showed a nearly threefold increase in the risk of colorectal cancer compared with those from families reporting the lowest intake. The elevated risk remained even after the researchers adjusted the data for potential confounders such as socioeconomic status and meat, fruit, and vegetable intake. No other cancers were significantly affected by higher dairy intakes.

“The mechanisms underlying these associations remain unknown, but there is increasing evidence that nutrition early in life can have long-lasting programming effects,” says lead author Jolieke C. van der Pols, an epidemiologist at the University of Queensland. For example, childhood dairy intake appears to be inversely associated with adulthood concentrations of insulin-like growth factor 1 (IGF-1), a key player in the development and progression of colorectal cancer. But it’s the effect of early dairy intake on childhood (rather than on adult) concentrations of IGF-1 that may be the important mediator of colorectal cancer risk.

The findings seem perplexing given previous research showing an association between high dairy/calcium consumption and lower risk of colorectal cancer in adulthood. “In adults, this protection occurs despite the increase in growth factors, which would be expected to increase risk,” says epidemiologist Edward Giovannucci of the Harvard School of Public Health. “It is possible, though not proven, that the increase in growth factors early in life may be more important for colorectal cancer risk.” Giovannucci asserts that the new findings are biologically plausible and warrant efforts to replicate these findings in other populations and settings.

Andrew Szilagyi, a gastroenterologist and assistant professor of medicine at McGill University School of Medicine, points out the lack of data on adult dietary intakes in the Boyd Orr cohort. “We do not know any aspects of dietary intake in adulthood,” Szilagyi says. “Nor do we know that the adults who developed colorectal cancer were also the very children in the families that indeed had higher dairy intakes.” In light of the fact that most studies have reported a protective effect of dairy products, it is important to determine the extent to which the former diet was continued into adulthood, he notes.

The milk consumption levels identified as posing a significant cancer risk in the Boyd Orr cohort are similar to current average intakes for U.S. children. Nonetheless, the researchers assert that it would be premature to consider altering current guidelines for children’s nutrition. “Dairy products are important contributors to children’s intake of protein, vitamins, and minerals,” says van der Pols. “Because this is only the first study to show associations between childhood dairy consumption and risk of cancer in adulthood, more evidence is needed before any firm conclusions can be drawn.”

